# Supplementary material for: Protamine Characterization by Top-Down Proteomics: Boosting Proteoform Identification with DBSCAN
Source: Proteomes. 2021 Apr 30;9(2):21. doi: 10.3390/proteomes9020021 (PMC8162566; doi:10.3390/proteomes9020021)
Supplement: Supplementary file 1 [file proteomes-09-00021-s001.zip › Arauz_Proteomes_2021_SupFig.pdf]

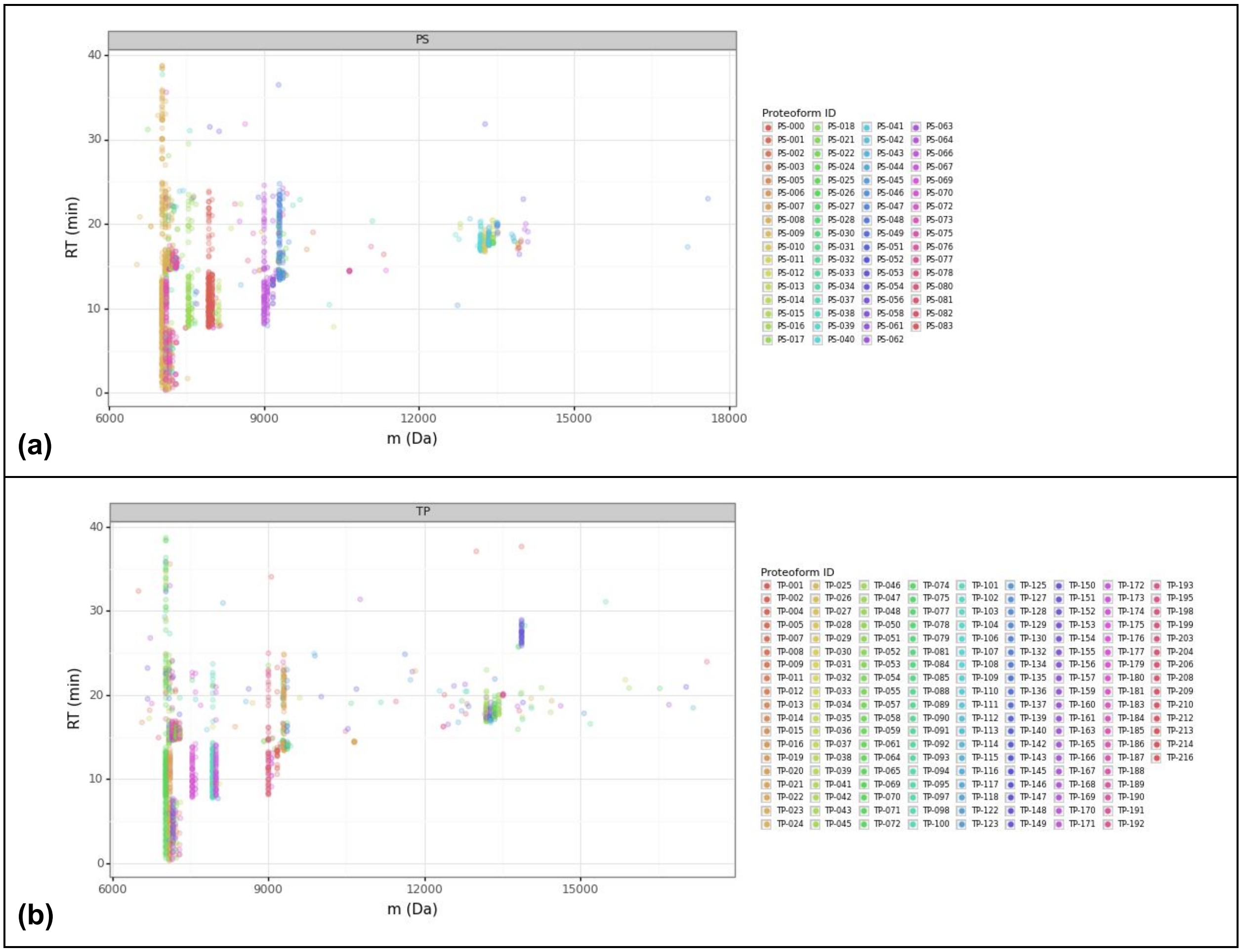

Figure S1. Scatterplots of the retention time (*RT*) vs. the deconvoluted experimental mass (*m*) by proteoform ID. The sequential color scale highlights the proteoform IDs assigned to each PrSM according to a) PS or b) TP.

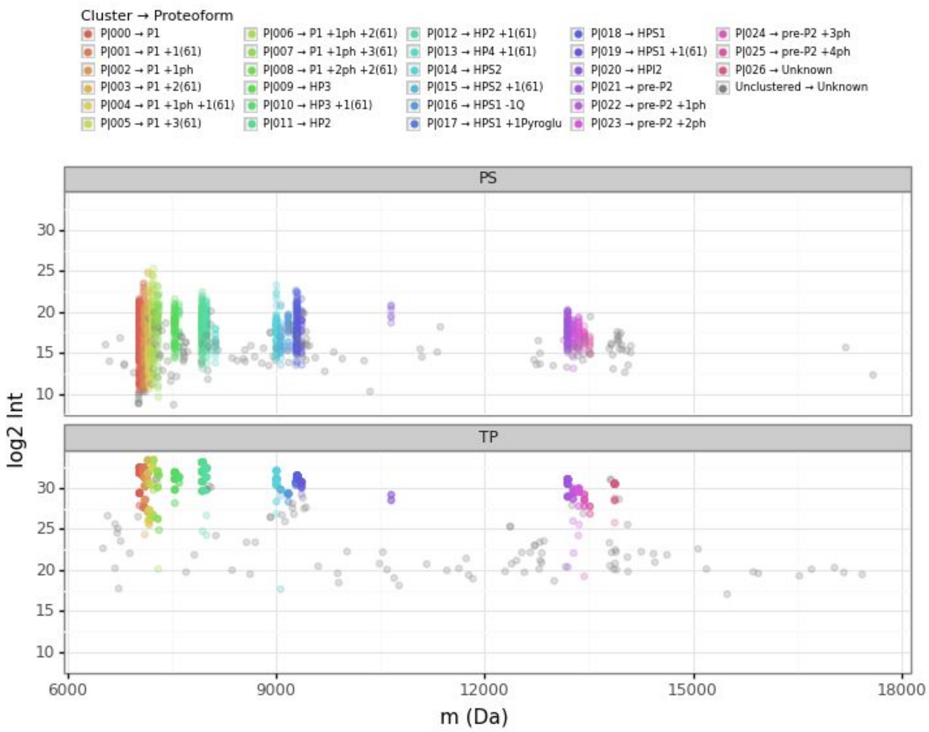

(a)

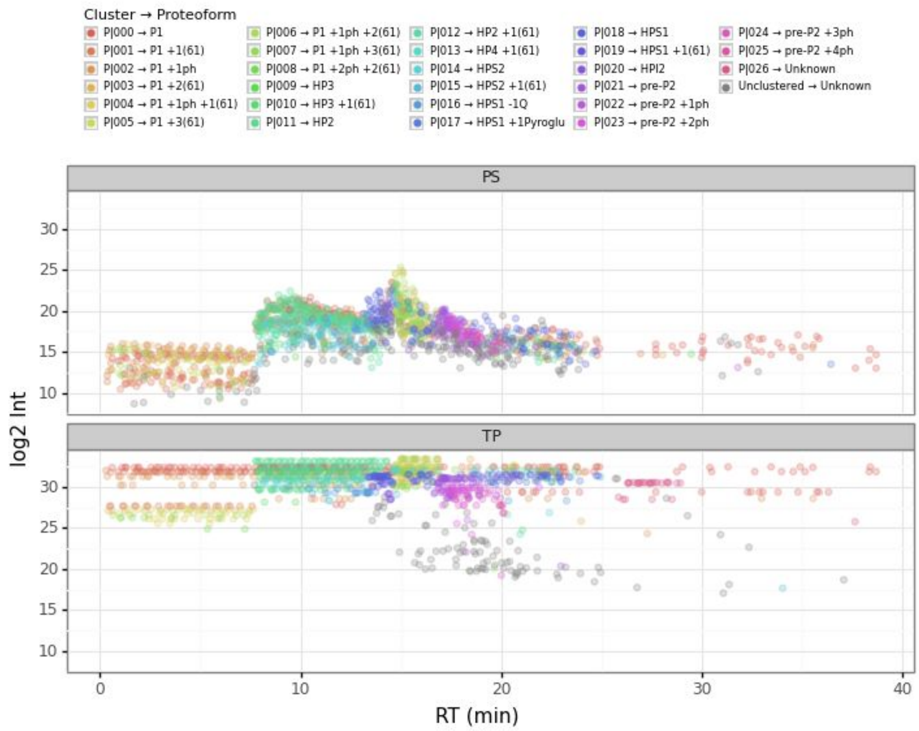

(b)

Figure S2. Scatterplots of the intensity (*log2 Int*) vs. the deconvoluted experimental mass (*m*) and the retention time (*RT*) for PS and TP by cluster. The sequential color scale highlights the clusters obtained sorted by increasing experimental mass, while unclustered PrSMs appear in grey. a) *log2 Int* vs. *m* projection. b) *log2 Int* vs. *RT* projection. (ph = phosphorylation, Q = glutamine, pyroGlu = pyroglutamic acid).

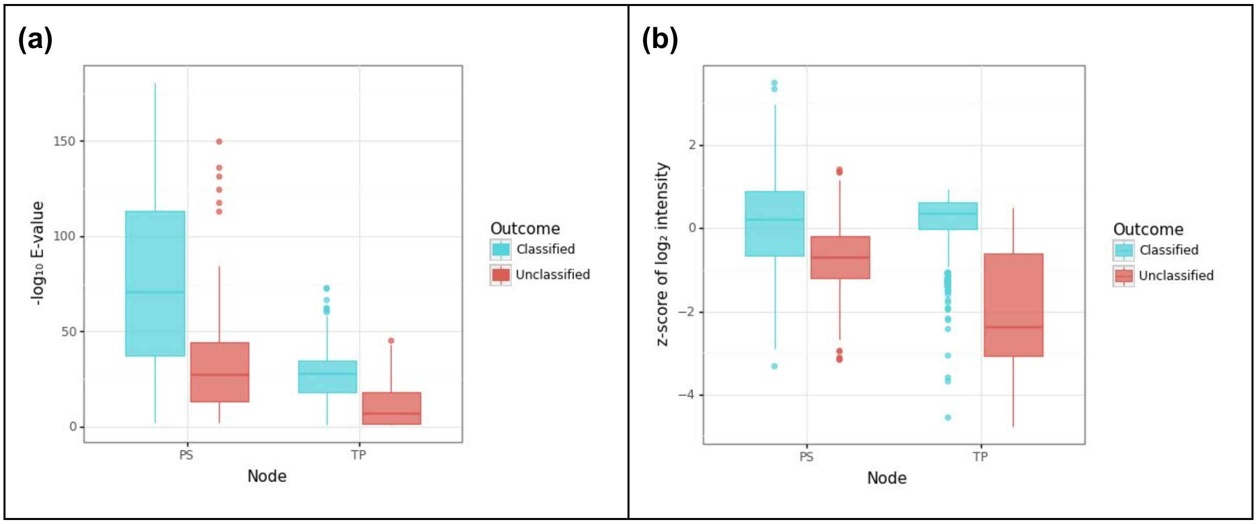

Figure S3. Boxplot of -log$_{10}$ E-value and z-score of log$_2$ intensity vs. Node by outcome (classified and unclassified PrSMs).
